# New Strategies for Treating Myomas

**DOI:** 10.1155/DTE.2.129

**Published:** 1996

**Authors:** Carl Wood, Peter Maher

**Affiliations:** 1 Melbourne Gynoscopy Centre Department of Obstetrics and Gynaecology Monash University Melbourne Victoria Australia; 2 Endosurgical Unit Mercy Hospital for Women Melbourne Victoria Australia; 3 The Melbourne Gynescopy Centre 284 High St. Asburton Victoria 3147 Australia

## Abstract

Laparoscopic minilaparotomy in 6 patients using the Maher abdominal elevator facilitated both quicker enucleation and morcellation of the myoma and suture of the myoma cavity. Myoma reduction in 12 patients by electrosurgery resulted in a 60% reduction in myoma diameter with failure in 2 patients. This technique may avoid myomectomy and be particularly useful in patients with infertility or near menopause.


*Diagnostic and Therapeutic Endoscopy*, 1996, Vol. 2, pp. 129–134
Reprints available directly from the publisher
Photocopying permitted by license only

© 1996 OPA (Overseas Publishers Association)
Amsterdam B.V. Published in The Netherlands
by Harwood Academic Publishers GmbH
Printed in Singapore

### New Strategies for Treating Myomas

CARL WOOD and PETER MAHER

*Melbourne Gynoscopy Centre, Department of Obstetrics and Gynaecology, Monash University, and Endosurgical Unit, Mercy Hospital for Women, Melbourne, Victoria, Australia*

*(Received June 28, 1995; in final form August 7, 1995)*

Laparoscopic minilaparotomy in 6 patients using the Maher abdominal elevator facilitated both quicker enucleation and morcellation of the myoma and suture of the myoma cavity. Myoma reduction in 12 patients by electrosurgery resulted in a 60% reduction in myoma diameter with failure in 2 patients. This technique may avoid myomectomy and be particularly useful in patients with infertility or near menopause.

**KEY WORDS:** Myoma, minilaparotomy, myolysis.

### INTRODUCTION

Myomas may require removal because of menstrual symptoms or infertility when the myoma is in the uterine cavity or blocking a tube, causing abdominal pain, pressure symptoms on the bladder, or rectum or when they are excessively large and cause abdominal distention. Myomas have been treated by myomectomy and/or shrinkage with gonadotropin-releasing hormone (GnRH) analogs. Myomectomy may be associated with significant blood loss (500 ml on average), the risk of blood transfusion (41%), or the need to proceed to undesired hysterectomy because of difficulty controlling hemorrhage (1). Adhesions may occur in 40% or more of patients having myomectomy, which may cause infertility or pain (2).

GnRH analogues are used to shrink myomas, the reduction being up to 40 to 50% after 12 weeks' healing (3). Regrowth usually occurs within 6 to 12 months of cesation of therapy except in postmenopausal women (3). Hormone replacement therapy in small doses reduces the side effects of the drugs (4). Despite the temporary effect of drug therapy, preoperative GnRH analogues have proven useful before myomectomy by reducing menstrual blood loss, increasing hemoglobin level, reducing operative blood loss by up to 40%, reducing the use of blood transfusion, making operative removal easier, and increasing the chance of laparoscopic removal (3). Nevertheless, blood loss at myomectomy after GnRH is still nearly twice as large as at hysterectomy and may be a cause for concern (3).

The frequent occurrence of adhesions after myomectomy has not been overcome by the use of Hyskon or Interceed (5). Nezhat et al (6) demonstrated few adhesions (3%) when the myoma cavity was not sutured, compared with 45% when the cavity was sutured. The myoma cavities sutured may have been larger than those not sutured in this study. The absence of sutures may lead to indentations and weakness of the uterine scar. The frequency of conception in 1,143 women wanting children after myomectomy was only 40%, suggesting that postoperative adhesion formation may be one factor limiting their fertility (2).

We have used two new techniques to deal with myomas—myoma reduction and laparoscopic minilaparotomy—to facilitate minimally invasive surgery for myomectomy and myoma reduction.

### LAPAROSCOPIC MINILAPAROTOMY FOR MYOMECTOMY

The lack of acceptance of laparoscopic surgery for more complex surgical procedures may be due to many factors: the lack of manual contact with anatomical and pathological structures, the longer training period required to learn the techniques, the loss of visual depth perception,

Address correspondence to: Prof. Carl Wood, Gynecologist, The Melbourne Gynescopy Centre, 284 High St., Asburton, Victoria 3147, Australia.

the need for video-based brain-hand coordination, the increased cost of equipment, and the prolongation of operations such as myomectomy and hysterectomy.

Laparoscopic minilaparotomy has been developed to combine the advantages of laparoscopy and laparotomy (7). It depends on the use of an abdominal elevator, (e.g.), the Maher elevator, and a 2-cm low abdominal transverse incision. This facilitates access for laparotomy instruments, finger palpation and dissection; binocular vision; and the avoidance of raised intra-abdominal pressure, which may predispose to cardiopulmonary compromise, gas embolus, or thromboembolism. The use of laparotomy instruments and finger palpation may also assist surgeons who find advanced laparoscopy techniques difficult.

The disadvantages of minilaparotomy are the two 6-mm incisions required to insert the elevator, the extension of the midline suprapubic incision to 2 or 3 cm, and the cost of the Maher abdominal elevator. There have been no hematomas in placement of the abdominal elevator in the lower abdomen after the site of insertion was standardized. The incisions are made in the thinnest part of the lower left abdominal wall, lateral to the inferior epigastric vessels and 2 to 3 cm medial to the anterior superior iliac spine, which avoids the superficial circumflex iliac vessels. The midline transverse suprapubic incision is made at least 3 cm below the insertion of the abdominal elevator. The rectus sheath is incised transversely, and the rectus muscle is split, or if thick and impeding retraction, cut by electrosurgery. This incision is kept open either by narrow curved metal retractors, by a 2- to 3-cm length of a 20-ml plastic syringe barrel, or by suturing the peritoneum to the abdominal skin.

The minilaparotomy incision does not delay recovery when compared with the usual 0.5- to 1.0-cm laparoscopic incisions as the former bruises less than the umbilical puncture site, which involves a blind puncture that traverses thick rectus muscle. The minilaparotomy incision site is in the nonmoving part of the abdomen, which reduces stretching during recovery. Some laparoscopic techniques utilize 2-cm incisions usually created by blind dilatation and tearing of a 1-cm incision.

The Maher abdominal elevator costs only $600 and is made by Cook (Australia). The sharp end is replaceable, and the main bar is robust. There is a safety shield that covers the tip. The sharp tip is watched during insertion as the safety guard only covers the tip after insertion. The purpose of the shield is to guard against accidental perforation of tissue when passing the elevator from the entry wound on the one side of the abdomen to the exit wound on the other. No complications have been associated with the use of the elevator. Other abdominal elevators are effective. The laparofan can be placed before a pneumoperitoneum is made, but the cost of the elevator is several thousand dollars.

One disadvantage associated with the open port of a minilaparotomy is that bowel tends to prolapse into the pelvis because of the absence of raised intra-abdominal pressure. This may necessitate the use of an extra incision to insert a forceps capable of safely retracting the bowel, and a fan-shaped forceps is available for this purpose, or a narrow pack, which can traverse the 2- to 3-cm incision can be inserted.

### Subjects and Methods

Minilaparotomy was used in association with the Maher abdominal wall elevator in 6 patients each with one to four myomas from 5 to 10 cm in diameter. The capsules of the myomas were incised transversely by a spoon electrode carrying 70 watts cutting monopolar current. The myoma was stabilized by a 5-mm diameter myoma screw. The forefinger was inserted through a 2- to 3-cm lower abdominal incision made over the maximum curvature of the surface of the myoma. The myoma was enucleated by finger dissection (Fig. 1A), and hemostasis was ensured by bipolar forceps coagulation. The myoma was cut into pieces by a scalpel and scissors inserted into the abdomen through the same incision, and the pieces of myoma were further reduced in size by morcellation with scissors as they were pulled through the incision (Fig. 1B). The myoma cavity was explored by the finger and sutured intraperitoneally by a size 1 vicryl suture held in a long-handled needle holder normally used at laparotomy, the suture line being visible both through the incision and the laparoscope (Fig. 1C). The uterus was held steady by a blunt curette inside the cavity, and the edges of the suture line were manipulated by laparoscopically placed grasping forceps.

### Results

The operative time in the 6 patients was between 30 and 60 minutes. The patients having a laparoscopic myomectomy were discharged home the next day and had no postoperative complications. The 2- to 3-cm incision did not delay recovery in the hospital or at home.

### Discussion

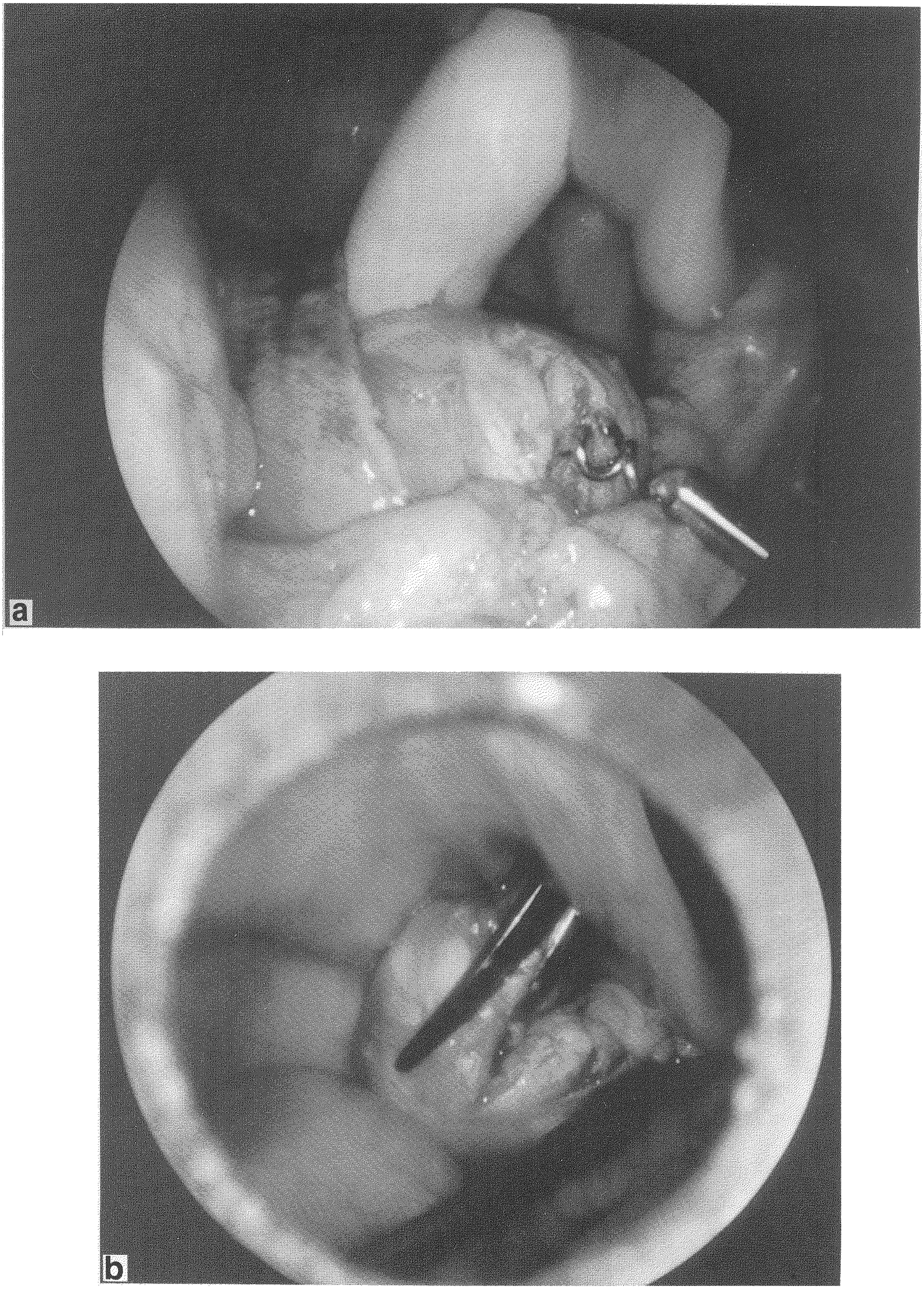

An advantage of the finger dissection when compared with instrumental operative laparoscopy techniques was the speed in enucleation of the myoma. Another advantage of mechanical elevation of the abdominal wall was that the incision could be used as a port for long-handled laparotomy instruments. Morcellation and suturing can then be a
b

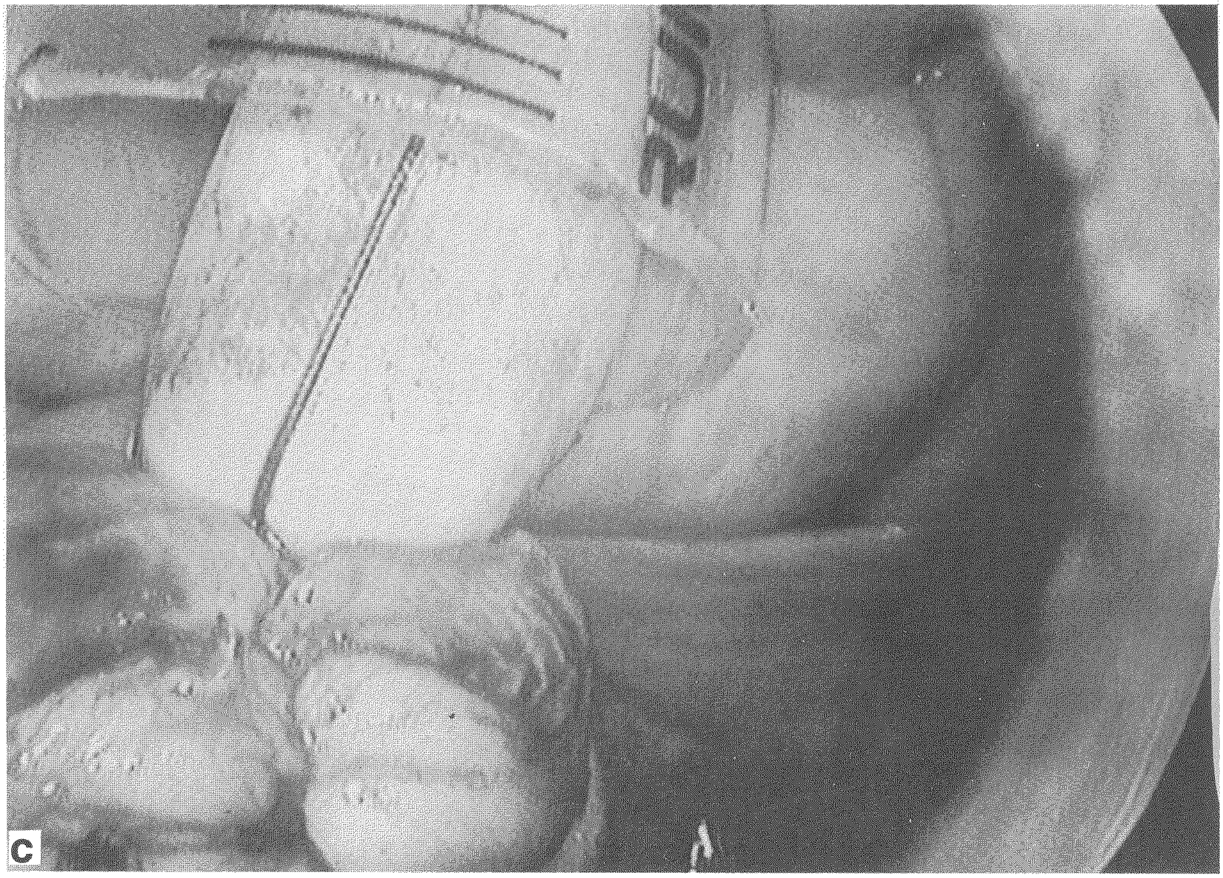

**Figure 1** A. Finger enucleation of myoma through minilaparotomy incision. B. Morcellation of myoma by scissors or scalpel. C. Suture of myoma capsule and finger tie of knot through minilaparotomy self-retaining retractor (syringe barrel). (Figure reproduced with permission from Blackwell Scientific Publications Ltd.)

performed under laparoscopic control or by observation through the open port, assisted by binocular vision.

### MYOMA REDUCTION BY ELECTROSURGERY

Goldfarb (8) demonstrated that coagulation of myomas by Nd:YAG laser may reduce the size of myomas by 50% or obliterate myomas of 2 to 3 cm. This surgery was done in women who were near menopause, and no regrowth was noticed over 14 months. The current study explored the use of coagulative electrocautery in 12 patients with myomas. We reported the successful use of electrosurgery in reducing myomas in 4 patients (9).

### Subjects and Methods

Twelve patients with 32 subserous or intramural myomas were selected for the procedure, 10 with menorrhagia and 2 with infertility only. Two of these patients were in the previous study. All had pre- and postoperative vaginal ultrasonography (Table 1). Hysteroscopy was carried out before operative laparoscopy to exclude the presence of intracavity myomas.

The operation was performed by laparoscopy, one accessory incision in the lower abdomen being for a myoma screw holding the myoma and another accessory incision being for a monopolar electro-surgical needle, the Goldfarb bipolar needle or suction irrigator.

The myoma was first injected with 1 in 50 vasopressin and the myoma was electrocoagulated using a monopolar electrosurgical needle and a 50-watt coagulation current or the bipolar needle with a 100-watt cutting current. Both needles had 4- to 5-cm bare tips, so care to avoid contact burns during use was required. A high-density current is necessary because the long length of the needle disperses more current.

The needle was inserted deep into the myoma at 1-cm intervals over its surface. Blanching occurred up to a 1-cm distance from the point of needle insertion (Fig. 2).

The myometrium next to the myoma, in a direct line from the ascending uterine vessels, was also coagulated. One or two liters of a buffered solution (Ringer's or Hartmann's) was left in the peritoneal cavity at the completion of the operation.

### Results

The results of the procedures are shown in Table 1. As mentioned, menstrual symptoms were the indications for myoma reduction in 10 patients and in the other 2 patients the myoma was an incidental finding in young women with infertility.

There was no significant bleeding except in the 1 patient who also had a myomectomy and lost 150 ml of blood. Laparoscopy in 5 patients between 3 and 12 months subsequent to surgery showed that only 1 patient had adhesions, and these were related to the myomectomy scar not to the site of electrocoagulation. Postoperative pain lasted only 1 to 3 days, and there were no complications.

The two patients with persistent menorrhagia had to have hysterectomies.

**Table 1** Clinical Characteristics of 10 Menorrhagic Patients

| | |
|---|---|
| Age of patients | 27–42 yr |
| Preoperative diameter of myomas | 3–7 cm |
| Postoperative diameter of myomas | 0–4 cm |
| Percentage reduction in diameter | 60% (0–100%) |
| Relief of menorrhagia | 8/10 |
| Length of follow-Up | 6–36 months |

### Discussion

The successful electrocoagulation of myomas between 3 and 7 cm in diameter suggests that this may be a suitable method for their treatment. The range of reduction was large. The two largest myomas, 6 and 7 cm, both shrunk to 2 and 3 cm, respectively, and regrowth in 5 patients followed for 2 or more years has been small, 0 to 1 cm. All the myomas successfully treated were both subserous and intramural. The 4-cm myoma unsuccessfully treated was intramural. It may be difficult to target myomas that are not protruding from the uterine surface.

Needle bipolar forceps may have the advantage of precise control of the area of electrocoagulation although the bipolar needle prongs tended to bend toward each other on penetration of the myoma.

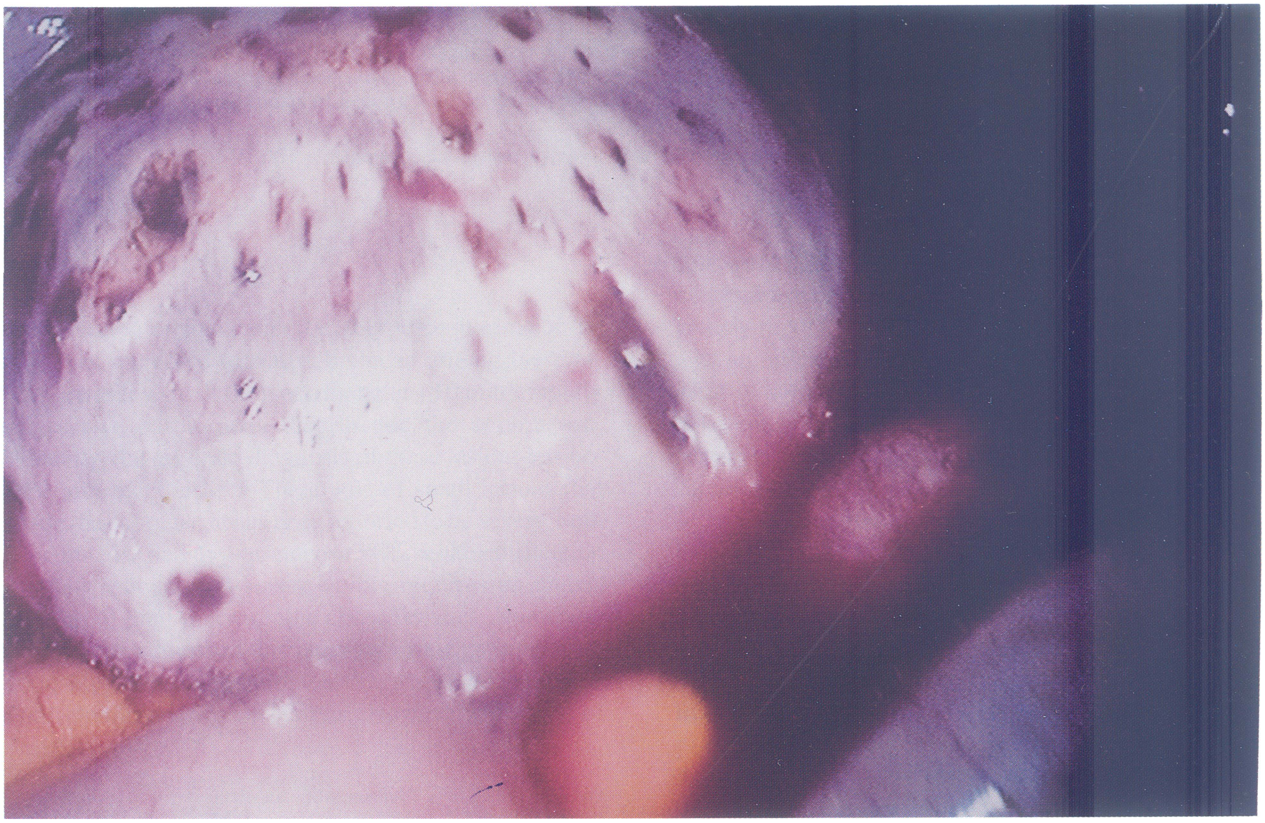

**Figure 2** Electrocoagulation of subserous myoma.

Vasopressin injection into the uterus has been associated with bradycardia and hypertension in France and is no longer used there (J. P. Pouly, personal communication). Electrocoagulation may assure hemostasis without the use of vasopressin.

The current study excluded myomas larger than 7 cm because of the possibility that necrosis of large volumes of tissue may result in absorption of necrotic tissue into the circulation, with uncertain side effects. Goldfarb (8,10) reduced the size of larger myomas up to 10 cm. It is still uncertain whether surgical reduction would lead to permanent shrinkage; Goldfarb's patients were close to menopause when myoma regrowth is also less common after the use of GnRH analogues. The GnRH analogues cause cell shrinkage, not cell reduction, and this is why regrowth occurs when estrogen levels return to normal.

Surgical reduction by cautery or laser causes cell necrosis so that myoma reduction may be permanent. Goldfarb has found little regrowth after 3 years. The scar formed at the surgical site of electrocoagulation appeared healthy, and myometrial thickness was not reduced. However, the strength of the scar cannot be estimated.

Further testing of electrocoagulation in the treatment of subserous myomas up to 8 cm diameter is suggested. The possible advantages may be reductions in blood loss, in the risk of blood transfusion, in the risk of necessity for hysterectomy, and in postoperative adhesive formation.

### REFERENCES

1. Gardner RL, Shaw RL. GnRH agonists and blood loss at surgery. In: Shaw R, ed. Uterine fibroids: a time for review. Carnforth, UK: Parthenon Publishing, 1991:123–124.
2. Buttram VC, Reiter RC. Uterine leiomyoma: etiology, symptomatology and management. Fertil Sterility 1981;36:433–437.
3. Shaw RW. Mechanism of action of GnRH agonists in the treatment of uterine fibroids. In: Shaw R, ed. Uterine fibroids: a time for review. Carnforth, UK: Parthenon Publishing, 1992;113–122.
4. Schweppe K-W. GnRH analogues in the treatment of uterine fibroids: results of clinical studies. In: Shaw R, ed. Uterine fibroids: a time for review. Carnforth, UK: Parthenon Publishing, 1992:103–111.
5. Surrey C. Adhesive formation after myomectomy. Abstract from the Biennial Meeting of International Society of Gynaecological Endoscopists, Washington, July 1993.
6. Nezhat C, Nezhat F, Silfen S, et al. Laparoscopic myomectomy. Int J Fertil 1991;36:275–280.
7. Maher P, Wood C, Hill D. Endoscopic minilaparotomy. Aust NZ J Obstet Gynaecol 1995;35:76–78.
8. Goldfarb HA. Nd:YAG laser laparoscopic coagulation of symptomatic myomas. J Reprod Med 1992;37:636–638.
9. Wood C, Maher P, Hill D. Myoma reduction by electrocautery. Gynaecol Endosc 1994;3:163–165.
10. Goldfarb HA. Electrocoagulation of myomas. Abstract from the Biennial Meeting of the International Society of Gynaecological Endoscopists, London, May 1–3, 1995.

